# Lipid droplet–mitochondria crosstalk in osteoarthritis: linking lipid routing to metabolic mismatch

**DOI:** 10.3389/fmolb.2026.1973436

**Published:** 2026-10-05

**Authors:** Gang Meng, Ruijie Hou, Ping Dong, Qiannan Chen, Xueyuan Li, Kai Hu, Zixuan Du, Rong Wang, Zhaoxin Zhong

**Affiliations:** 1 Inner Mongolia Medical University, Hohhot, Inner Mongolia, China; 2 Changchun University of Chinese Medicine, Changchun, Jilin, China; 3 Shanxi University of Traditional Chinese Medicine, Jinzhong, Shanxi, China

**Keywords:** fatty acid oxidation, ferroptosis, lipid droplets, lipid flux, mitochondrial metabolism, mitophagy, organelle contact sites, osteoarthritis

## Abstract

Osteoarthritis (OA) is a whole-joint disease characterized by metabolic reprogramming beyond structural degeneration. Increasing evidence indicates that lipid droplets (LDs) regulate lipid storage, mobilization and intracellular lipid routing, while alterations in mitochondrial metabolism determine the capacity of chondrocytes to adapt to metabolic stress. However, LD accumulation alone is insufficient to define metabolic dysfunction, as it may represent either adaptive buffering or maladaptive retention. This review proposes a lipid flux–mitochondrial capacity matching framework to integrate lipid handling with mitochondrial oxidative capacity, redox regulation and quality control. We discuss how excessive lipid flux relative to mitochondrial capacity may promote oxidative stress, lipid peroxidation, ferroptosis, senescence and extracellular matrix disruption, whereas balanced lipid utilization supports chondrocyte homeostasis. Current evidence on LD biology, fatty acid oxidation, mitophagy and whole-joint metabolic communication is summarized, with clear distinction between direct OA evidence and findings from other cellular systems. Although organelle contact sites such as PLIN5–FATP4, PLIN3–VDAC3 and ESYT1/2–VAPB represent potential regulators of lipid routing, their functional relevance in OA chondrocytes remains to be established. Future studies integrating isotope tracing, metabolic flux analysis and dynamic organelle imaging are required to clarify how lipid routing and mitochondrial capacity jointly regulate OA progression.

## Graphical Abstract

**Figure:**
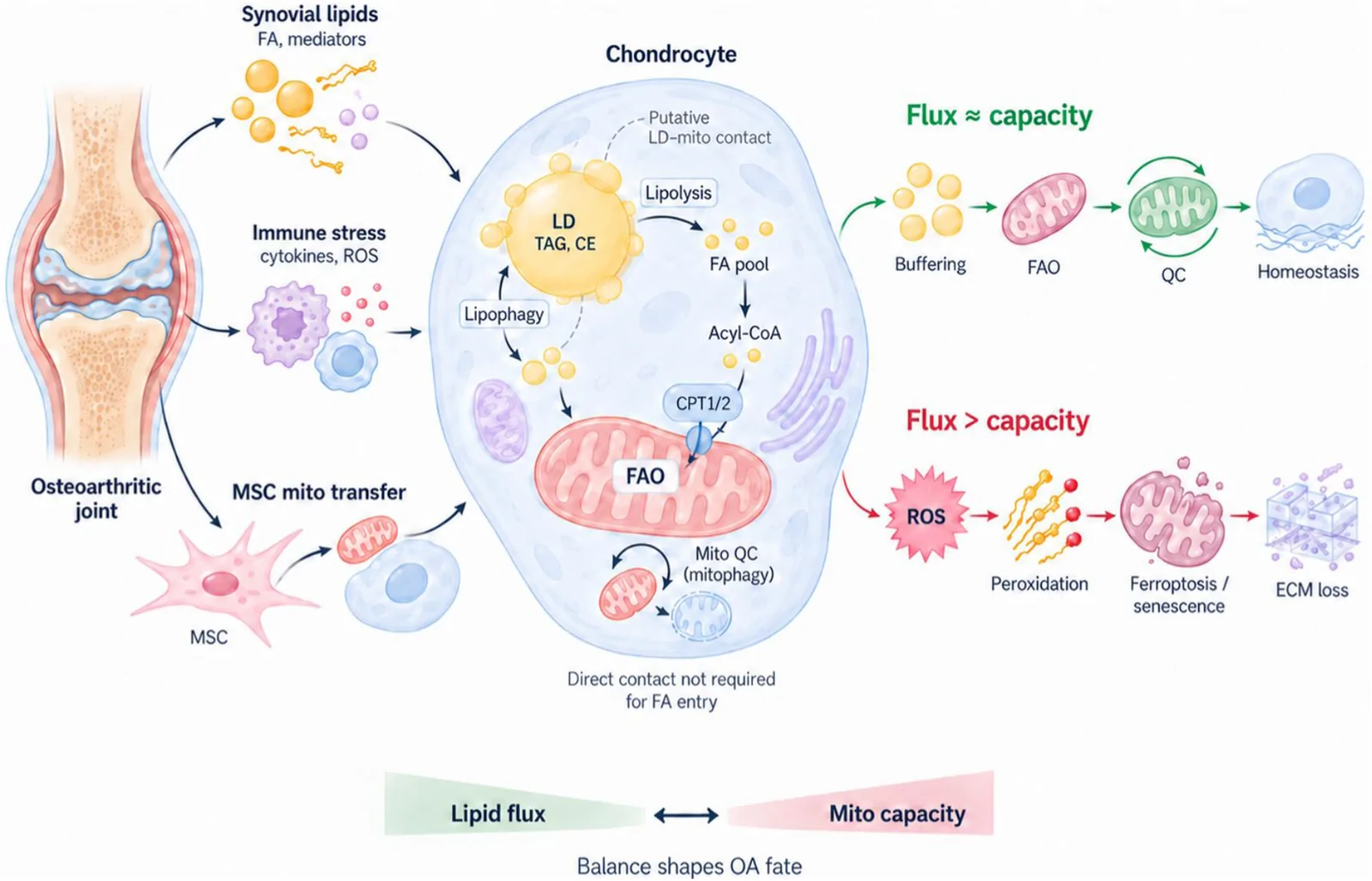

## 1 Introduction

Osteoarthritis (OA) is a heterogeneous whole-joint disease involving articular cartilage, synovium, subchondral bone and associated stromal and immune compartments (Wang et al., 2022; Tang et al., 2024; Raut et al., 2025). Although traditionally considered a degenerative disorder driven by mechanical stress and extracellular matrix breakdown, increasing evidence indicates that OA progression is accompanied by extensive metabolic reprogramming, including alterations in lipid metabolism, mitochondrial function and cellular stress adaptation. Lipid dysregulation in OA therefore extends beyond simple lipid accumulation and involves coordinated changes in lipid uptake, synthesis, storage, mobilization and utilization. However, how intracellular lipid handling is integrated with mitochondrial metabolic capacity to determine chondrocyte fate remains insufficiently understood.

Lipid droplets (LDs) represent dynamic intracellular organelles that regulate lipid storage and mobilization rather than passive lipid deposits. Recent studies have demonstrated that OA-associated LD accumulation may arise from distinct mechanisms, including impaired lipid droplet trafficking and lysosomal clearance under cellular stress (Yu et al., 2025), enhanced fatty acid synthesis and altered lipid metabolic programming (Mei et al., 2025), and broader remodeling of intracellular lipid composition (Xu K. et al., 2026). Importantly, LD accumulation alone cannot determine whether cells undergo metabolic adaptation or lipotoxic injury, because LD formation may serve as a protective buffering mechanism by temporarily sequestering excess fatty acids, whereas defective turnover or inappropriate lipid routing may contribute to pathological stress. Therefore, understanding OA-associated lipid abnormalities requires consideration of lipid composition, turnover and metabolic fate in addition to LD abundance.

The functional consequence of altered lipid availability depends largely on mitochondrial capacity, including oxidative substrate utilization, redox buffering and mitochondrial quality-control mechanisms. Fatty acids mobilized from intracellular lipid pools may undergo mitochondrial oxidation, re-esterification, membrane lipid synthesis or signaling pathways, and their biological effects are determined by whether mitochondrial processing capacity can accommodate metabolic demand. In OA chondrocytes, enhanced fatty acid oxidation promotes acetyl-CoA/HADHA-dependent metabolic reprogramming, resulting in SOX9 destabilization and cartilage catabolic changes (Mei et al., 2025). Conversely, restoration of mitochondrial quality control through HCAR2/AMPK/PINK1/Parkin-mediated mitophagy alleviates cartilage degeneration (Zhuang et al., 2024). These findings suggest that mitochondrial dysfunction in OA should not simply be defined as reduced mitochondrial activity, but rather as impaired metabolic flexibility and insufficient adaptation to changing substrate availability.

Recent studies have further highlighted potential functional connections between LD metabolism and mitochondrial utilization. In non-chondrocyte systems, LD–mitochondria contact sites mediated by proteins such as PLIN5–FATP4 have been shown to facilitate fatty-acid transfer toward mitochondrial β-oxidation (Miner et al., 2023). However, whether similar contact structures exist, and whether they contribute to lipid routing in OA chondrocytes, remains unknown. Thus, LD–mitochondria interactions should be considered as a potential regulatory mechanism requiring direct validation in OA models rather than an established disease mechanism. Meanwhile, impaired mitochondrial renewal and ferroptosis-related lipid remodeling have emerged as important contributors to OA progression (Cao S. et al., 2025; Deng et al., 2025), further supporting the concept that lipid handling and mitochondrial competence are functionally interconnected.

In this review, we propose a lipid flux–mitochondrial capacity matching framework to integrate lipid storage, mobilization, intracellular routing and mitochondrial metabolic regulation in OA. Rather than interpreting LD accumulation or fatty acid oxidation as inherently pathological or protective, this framework emphasizes the balance between lipid supply and mitochondrial processing capacity. We distinguish direct OA evidence from mechanistic findings obtained in other cellular systems and discuss how metabolic mismatch may contribute to oxidative stress, lipid peroxidation, ferroptotic susceptibility, cellular senescence and extracellular matrix disruption. This perspective provides a conceptual framework for understanding how adaptive lipid metabolism may transition into metabolic dysfunction during OA progression.

## 2 Literature search and evidence selection

Relevant literature was identified through targeted searches of PubMed and Web of Science Core Collection, focusing primarily on studies published between January 2021 and August 2026. Earlier landmark studies were additionally considered when they provided fundamental mechanistic insights into lipid droplet biology, mitochondrial regulation, fatty acid metabolism, or organelle communication.

The search strategy combined controlled vocabulary and free-text terms related to osteoarthritis, lipid metabolism, and mitochondrial biology. Representative terms included (osteoarthritis OR OA) AND (lipid droplet OR lipid trafficking OR lipidomic OR fatty acid oxidation OR cholesterol OR oxysterol) AND (mitochondrial respiration OR mitochondrial metabolism OR mitophagy OR organelle contact site). Additional searches incorporated related concepts, including ferroptosis, senescence, stable isotope tracing, and metabolic flux analysis, to capture emerging mechanisms linking lipid handling with mitochondrial adaptation.

This review considered evidence from multiple experimental contexts, including human OA tissues, primary and established chondrocyte models, cartilage explants, experimental OA models, and defined non-chondrocyte systems. Findings from human tissues and OA-related models were considered direct evidence for disease relevance, whereas studies from other cellular systems, including fibroblasts, macrophages, hepatocytes, myocytes, and mesenchymal stromal cells, were interpreted as mechanistic references that may inform hypotheses but require validation in OA-specific contexts. Particular emphasis was placed on studies providing functional evidence through genetic or pharmacological manipulation, metabolic flux analysis, isotope tracing, or direct assessment of mitochondrial activity, while descriptive studies were used to support biological context and emerging concepts. Studies were screened based on title/abstract relevance followed by full-text assessment. Studies lacking mechanistic relevance to lipid handling, mitochondrial metabolism, or OA-associated metabolic regulation were excluded.

## 3 Lipid droplet homeostasis in osteoarthritis: from adaptive buffering to maladaptive retention

OA-associated LD accumulation is mechanistically heterogeneous, arising from increased lipid supply, enhanced *de novo* lipogenesis (DNL) or defective lipid handling (Figure 1). Importantly, OA-associated lipid remodeling extends beyond changes in LD abundance. Recent lipidomic analyses of human osteoarthritic cartilage have revealed disease-associated alterations across multiple lipid classes, including glycerolipids, cholesteryl esters, ceramides, sphingolipids and other complex lipid species, demonstrating that OA is characterized by broad remodeling of intracellular lipid composition rather than simple neutral lipid accumulation (Zhou et al., 2025). These findings highlight the importance of considering lipid composition, turnover and metabolic routing alongside LD abundance when interpreting OA-associated lipid alterations. In obesity-associated mouse cartilage, glucose and insulin drive ACC-dependent DNL (Liu et al., 2022), and loss of the PPARα–ACOT12 axis increases acetyl-CoA availability, promotes DNL and causes LD accumulation and cartilage catabolism in human OA chondrocytes and experimental OA (Park et al., 2022). LD changes in OA are therefore not a simple consequence of extracellular lipid supply.

**FIGURE 1:**
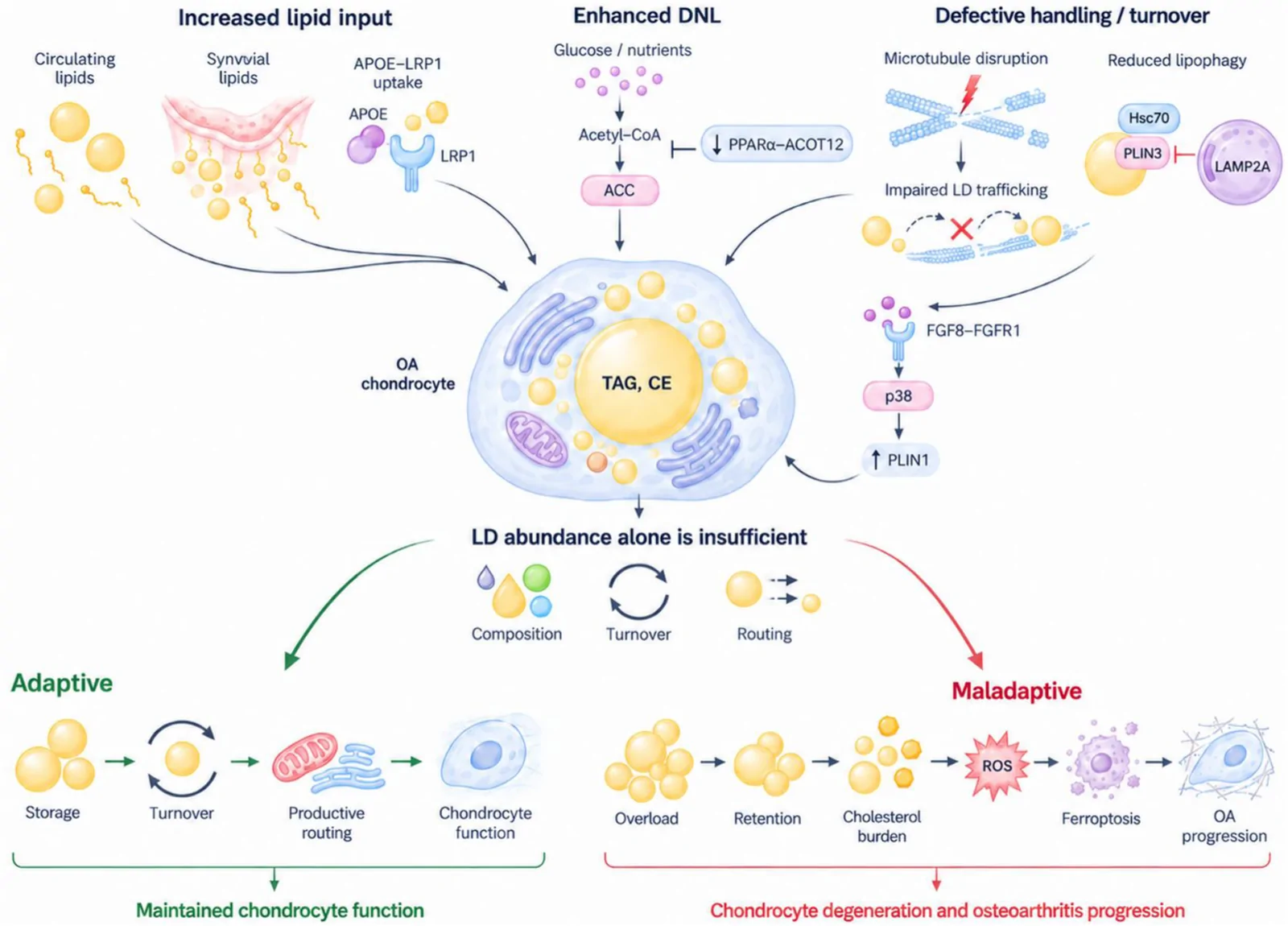
Dynamic dysregulation of lipid droplet homeostasis in osteoarthritis chondrocytes.

LD abundance is also regulated at the LD surface. FGF8–FGFR1–p38 signalling upregulates PLIN1 and increases LD accumulation in cultured chondrocytes (Huang et al., 2025), indicating that OA-relevant signalling alters the capacity to package neutral lipids, although the *in vivo* contribution of the FGF8–PLIN1 axis remains untested.

LD accumulation alone cannot indicate lipotoxicity or protection from it. LDs transiently buffer free fatty acids, and in high-fat-diet-associated OA mice and cultured articular chondrocytes, PKCK2–STAMP2–FSP27-dependent LD formation confers resistance to free fatty acid-induced lipotoxicity (Lee et al., 2018). This protection is not universal: metabolic tracing in human and murine OA articular chondrocytes shows simultaneous fatty-acid synthesis and oxidation (Wang et al., 2025a). Because distinct pools can be routed to storage, membrane synthesis or oxidation, the LD–FAO relationship requires direct inter-compartmental tracing. LD abundance alone is thus insufficient to define the underlying metabolic state, lipid composition, turnover, mobilization and routing may provide greater mechanistic insight.

Impaired LD turnover may also promote persistent accumulation. In stressed chondrocytes, microtubule disruption impairs LD trafficking, LD–lysosome interactions and Hsc70–PLIN3–LAMP2A-dependent lipophagic clearance, and microtubule stabilization restores clearance and phenotype (Yu et al., 2025). This study directly links turnover defects to LD retention but did not compare them with increased lipid input. The relative contribution of defective turnover *versus* enhanced supply therefore remains unresolved.

Redox regulation and cholesterol handling intersect with LD homeostasis. Swimming exercise increased cartilage SOD3 in OA mice, and SOD3–C/EBPβ interaction suppressed APOE transcription and APOE-dependent cholesterol uptake, reducing LD load and free cholesterol (Wu et al., 2025). Beyond cholesterol uptake and storage, oxysterol-mediated nuclear receptor signaling represents an additional layer of lipid regulation in osteoarthritis. Oxysterols, including 25-hydroxycholesterol and related cholesterol oxidation products, function as endogenous ligands of liver X receptors (LXRs), thereby regulating cholesterol efflux, lipid transport and inflammatory transcriptional programs. In OA-related contexts, altered cholesterol handling and LXR activity may influence chondrocyte lipid homeostasis. However, whether specific oxysterol species directly regulate LD remodeling or mitochondrial lipid utilization in OA chondrocytes remains insufficiently defined (Zhang Y. et al., 2025). Therefore, oxysterol–LXR signaling should be considered as a potential regulatory pathway linking cholesterol metabolism with broader lipid remodeling rather than as an established driver of LD accumulation. Because this study primarily demonstrates altered cholesterol uptake rather than lipid transport or clearance, broader conclusions on coordinated supply–transport–clearance changes require further evidence.

LD accumulation should not be viewed as intrinsically pathological. When supply remains within handling capacity, esterification into neutral lipids buffers excess fatty acids, whereas defective DGAT1-dependent biogenesis exacerbates lipotoxic stress and ferroptosis in other systems (Wang Y.-T. et al., 2025). Sustained lipid overload or turnover failure converts transient buffering into maladaptive retention, so the biological readout of LD abundance depends on lipid composition, remobilization and routing relative to mitochondrial capacity.

## 4 Lipid droplet–mitochondria interfaces and lipid routing in osteoarthritis

Fatty acids mobilized from LDs may be re-esterified, incorporated into membrane phospholipids, converted into signalling lipids or oxidized, we focus on oxidation. Mobilization itself uses distinct pathways: ATGL/HSL/MGL-dependent lipolysis releases fatty acids from triacylglycerols, whereas lipophagy delivers intact LDs to lysosomes. Both replenish the cellular fatty-acid pool but are differentially regulated, and neither implies global conversion of triacylglycerols to free fatty acids. After activation by acyl-CoA synthetases, mitochondrial entry proceeds mainly through the carnitine shuttle (CPT1/CPT2) and does not. In principle, require LD–mitochondria contact, such contacts could nevertheless concentrate this flow, and microtubule-dependent trafficking plus PLIN-dependent anchoring determine which LDs are accessible. In non-chondrocyte systems, PLIN5–FATP4 contacts channel LD-derived fatty acids to mitochondrial β-oxidation.

Miner et al. showed in myoblasts of human and murine origin that PLIN5–FATP4 binding channels LD-derived fatty acids to mitochondria for β-oxidation (Miner et al., 2023), and Wang et al. described in HEK293 and COS-7 cells a VPS13D–TSG101/ESCRT-dependent remodelling of LD membranes that facilitates transfer at mitochondria–LD contacts (Wang et al., 2021). Contact sites thus function as active lipid-transfer platforms in these systems.

Proximity proteomics in cultured cells identified an ESYT1–ESYT2–VAPB complex at LD–mitochondria–endoplasmic reticulum interfaces, its loss reduced LD-derived fatty-acid oxidation without altering physical proximity, dissociating contact abundance from transfer function (Bezawork-Geleta et al., 2025). No equivalent data exist for OA chondrocytes.

Contact does not necessarily commit lipids to oxidation. Loss of ESYT1, ESYT2 or VAPB limited LD-derived fatty-acid oxidation in cultured cells and Esyt1/Esyt2-deficient mice (Bezawork-Geleta et al., 2025), whereas PLIN5 phosphorylation in hepatocytes promoted esterification and storage (Kang et al., 2026). LD–mitochondria contacts are thus context-dependent routers rather than simple oxidation gates.

Tethering follows a metabolic logic and is tissue-specific. Energy stress activates Rab8a–PLIN5 tethering in rat L6 myotubes (Ouyang et al., 2023), and glucose starvation activates an AMPK–ARF1–PLIN2 axis in C2C12 myoblasts and mouse skeletal muscle (Chen L. et al., 2025). Structural contact abundance alone cannot predict transfer efficiency or downstream fate.

No functional LD–mitochondria contact has yet been reported in OA chondrocytes. Contact frequency, architecture and molecular identity across OA stages are unknown, and tethering machinery is tissue-specific, for example, Mfn2–Hsc70 in cardiomyocytes (Hu et al., 2024). LD–mitochondria contacts in OA are therefore a hypothesis to be tested rather than an established mechanism, and no assumption of tissue homology is warranted.

If such contacts exist in OA, their relevance lies in routing efficiency relative to mitochondrial capacity. Impaired mitophagy and mitochondrial dysfunction promote chondrocyte degeneration (Long et al., 2025), whereas BNIP3 activation restores mitophagy and limits cartilage damage (Gou et al., 2025). The cellular outcome depends on whether mitochondrial oxidative and quality-control systems accommodate incoming lipid flux (Figure 2).

**FIGURE 2:**
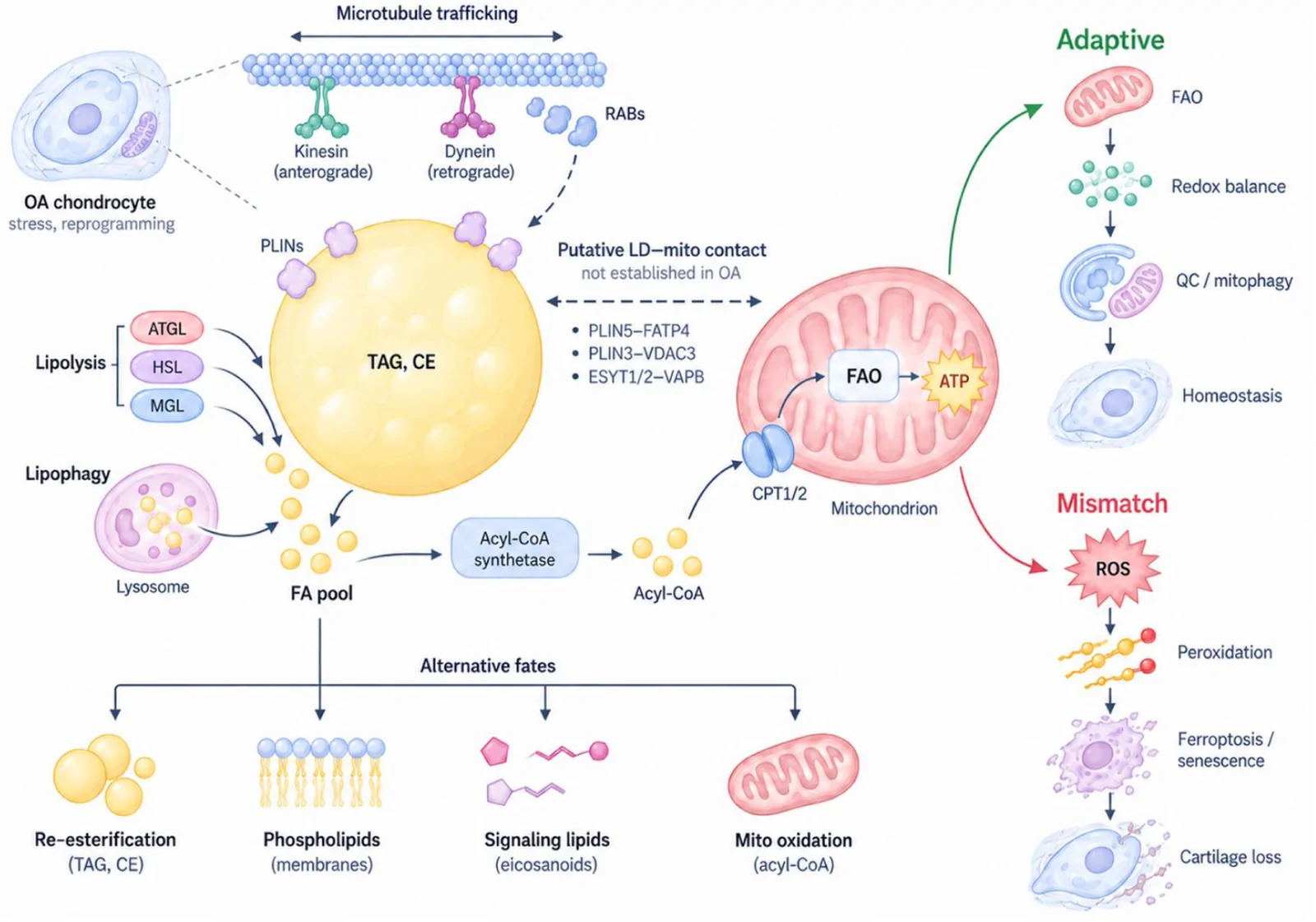
Context-dependent lipid routing at the lipid droplet–mitochondria interface in osteoarthritis.

Initial validation in OA chondrocytes: of the candidate systems above, three are prioritized. PLIN5–FATP4 has the strongest direct evidence of lipid transfer capacity; PLIN3–VDAC3 combines proximity proteomics with PLIN3 processing already implicated in stressed chondrocytes [4]; ESYT1–ESYT2–VAPB provides the clearest example of contact-independent transfer. Expression, localization, contact occupancy and lipid-transfer assays should be assessed in human OA cartilage first; VPS13D–TSG101 and Rab8a–PLIN5 are informative but lower priority because their OA relevance is untested. The current evidence for LD–mitochondria crosstalk and its potential relevance to OA is summarized in Table 1.

**TABLE 1:** Evidence framework for lipid droplet–mitochondria crosstalk: established mechanisms and potential relevance to osteoarthritis.

Mechanistic feature	Representative mechanism	Experimental context	Evidence status	Potential relevance to OA	Representative references
Contact–transfer dissociation	Physical proximity between lipid droplets (LDs) and mitochondria does not necessarily indicate functional fatty-acid transfer	Cultured cells; myoblasts; Esyt1/2-deficient mice (non-chondrocyte systems)	Indirect mechanistic evidence	LD–mitochondria contact abundance should be distinguished from actual lipid transfer activity in OA chondrocytes	(Bezawork-Geleta et al., 2025; Wang et al., 2021)
Context-dependent lipid routing	LD-derived fatty acids may undergo mitochondrial oxidation, re-esterification, membrane phospholipid synthesis or lipid signaling depending on cellular demand	Hepatocytes; skeletal muscle; cultured cells	Indirect mechanistic evidence	OA-associated lipid fate cannot be inferred from LD abundance or FAO alone	(Miner et al., 2023; Kang et al., 2026)
Tissue-specific tethering machinery	PLIN5–FATP4, ESYT1/2–VAPB and other tethering proteins regulate LD–mitochondria coupling in a tissue-dependent manner	Myoblasts, hepatocytes, cultured cells and mouse models	Established in non-cartilage systems	These proteins represent candidate regulators requiring validation in OA chondrocytes	(Miner et al., 2023; Wang et al., 2021; Bezawork-Geleta et al., 2025)
PLIN-dependent lipid transfer	PLIN5–FATP4 interaction facilitates LD-derived fatty-acid transport toward mitochondrial β-oxidation	Human and mouse myoblasts	Indirect mechanistic evidence	Provides a candidate mechanism for lipid routing, but its relevance to OA cartilage remains unknown	(Miner et al., 2023)
ESYT/VAPB-mediated contact regulation	ESYT1/2–VAPB complexes regulate LD–mitochondria–ER interfaces and influence lipid transfer efficiency	Cultured cells and mouse models	Indirect mechanistic evidence	Suggests that contact function rather than contact number may determine metabolic outcomes	Bezawork-Geleta et al., 2025
Candidate validation priorities in OA	PLIN5–FATP4, PLIN3–VDAC3 and ESYT1/2–VAPB represent candidate LD–mitochondria regulatory systems	Proposed validation in human OA chondrocytes	Hypothesis-generating	Requires spatial localization, genetic perturbation and direct lipid-transfer assays in OA models	(Miner et al., 2023; Bezawork-Geleta et al., 2025)

## 5 Mitochondrial capacity and lipid flux in osteoarthritic chondrocytes

To distinguish physiological metabolic adaptation from pathological metabolic mismatch, lipid–mitochondrial states in healthy and osteoarthritic chondrocytes are summarized in Table 2.

**TABLE 2:** Comparison of lipid–mitochondrial metabolic states between healthy and osteoarthritic chondrocytes.

Feature	Healthy cartilage	Osteoarthritic cartilage	Representative evidence
Lipid droplet biology	Dynamic lipid buffering, controlled neutral lipid storage and regulated turnover maintain intracellular lipid homeostasis	Increased lipid retention may result from altered lipid synthesis, trafficking or clearance; LD accumulation alone is insufficient to define the underlying metabolic state	(Yu et al., 2025; Zhou et al., 2025)
Lipid composition and remodeling	Balanced distribution of glycerolipids, cholesteryl esters, phospholipids and other lipid species supports membrane integrity and metabolic adaptation	OA involves broad lipid remodeling, including alterations in glycerolipids, cholesteryl esters, sphingolipids and other complex lipid classes	(Zhou et al., 2025)
Lipid routing	Coordinated partitioning of fatty acids among neutral lipid storage, membrane phospholipid synthesis, signaling pathways and mitochondrial oxidation according to cellular demand	Altered lipid partitioning may increase retention, redirect fatty acids toward oxidation or peroxidation, and disturb metabolic adaptation; the relative contribution of each route remains context-dependent	(Mei et al., 2025; Zhou et al., 2025; Wang et al., 2025a)
Fatty-acid utilization	Fatty-acid oxidation is matched with mitochondrial oxidative capacity and cellular energy requirements	Enhanced lipid flux or altered fatty-acid utilization may exceed mitochondrial processing capacity, contributing to metabolic stress and chondrocyte dysfunction	Mei et al., 2025; Wang et al., 2025a)
Mitochondrial oxidative capacity	Efficient substrate oxidation, ATP production and respiratory adaptation maintain metabolic flexibility under changing substrate availability	Reduced respiratory adaptability, impaired substrate utilization and decreased metabolic flexibility increase susceptibility to metabolic stress	(Liu et al., 2026)
Redox-buffering capacity	Controlled ROS production with effective antioxidant systems maintains redox balance	Increased mtROS accumulation, lipid peroxidation and impaired antioxidant responses contribute to oxidative damage and inflammatory signaling	(Wu et al., 2025; Jiang et al., 2025)
Mitochondrial quality-control capacity	Coordinated mitophagy, mitochondrial dynamics and clearance of damaged organelles preserve mitochondrial integrity	Dysregulated mitophagy and mitochondrial dynamics promote accumulation of dysfunctional mitochondria and impair stress adaptation	(Zhuang et al., 2024; Fang et al., 2023; Hu et al., 2023)
Lipid flux–mitochondrial capacity relationship	Lipid input, storage, mobilization and mitochondrial processing remain balanced, supporting metabolic adaptation	Lipid supply and intracellular routing may exceed mitochondrial oxidative and quality-control capacity, leading to redox imbalance, ferroptotic susceptibility and senescence	(Mei et al., 2025; Cao S. et al., 2025; Liu et al., 2026; Zou et al., 2025)
Chondrocyte phenotype and ECM homeostasis	Maintained SOX9/COL2A1/ACAN expression and extracellular matrix synthesis preserve cartilage phenotype	Metabolic mismatch promotes anabolic–catabolic imbalance, senescence, ferroptotic vulnerability and extracellular matrix degradation	(Li H. et al., 2026; Jia et al., 2026; Fang et al., 2026)

### 5.1 Physiological baseline of lipid–mitochondrial homeostasis in healthy chondrocytes

In healthy chondrocytes, oxidative metabolism supports ATP production and redox balance while fatty-acid utilization stays matched to oxidative capacity, and quality control (mitophagy plus fusion–fission dynamics) removes damaged organelles; in OA this balance is disrupted. Consequently, higher FAO is not uniformly beneficial: FPH2-mediated inhibition of CPT1-dependent mitochondrial entry improves chondrocyte metabolism and cartilage regeneration *in vivo* (Chen et al., 2024), whereas PKM2 dimerization disrupts MFN1-dependent mitochondrial fusion and function (Liu et al., 2026). Whether lipid oxidation helps or harms therefore depends on mitochondrial competence rather than on the absolute rate of FAO.

### 5.2 Defining mitochondrial oxidative, redox and quality-control capacity in osteoarthritic chondrocytes

Mitochondrial function denotes the capacity to utilize substrates, produce ATP, handle redox and preserve organelle integrity; mitochondrial capacity comprises three measurable components, namely, (i) oxidative capacity (substrate oxidation, oxygen consumption rate, ATP production, respiratory reserve), (ii) redox-buffering capacity (mtROS generation and antioxidant systems maintaining redox balance), and (iii) mitochondrial quality-control capacity (mitochondrial dynamics, mitophagic flux, clearance of damaged mitochondria). Mitochondrial quality control specifically denotes mitophagy and dynamics; biogenesis and proteostasis lie outside our scope. Protein abundance, for example, PINK1, Parkin, SIRT3 or respiratory-chain subunits records regulation not function. Capacity must be measured by metabolic flux analysis, extracellular flux, ATP production, ROS/redox assessment and dynamic mitophagy readouts.

Recent studies in OA chondrocytes further indicate that mitochondrial dysfunction is not represented by a uniform reduction in mitochondrial activity. Altered mitochondrial homeostasis and impaired metabolic adaptation contribute to OA progression. Thus, mitochondrial dysfunction in OA should be interpreted as reduced adaptive capacity to accommodate changing substrate availability rather than simply decreased mitochondrial activity (Liu et al., 2026).

### 5.3 Lipid flux–capacity mismatch in osteoarthritic chondrocytes

Compensatory increases in oxidative metabolism help sustain ATP and redox balance early, but become inadequate as dysfunction progresses. PDK2 deficiency restored oxidative phosphorylation, NAD+/NADH and ATP while reducing ROS and senescence in experimental OA (Han et al., 2025), whereas loss of PDZK1 increased damaged mitochondria, ROS and senescence (Shao et al., 2024). Oxidative metabolism is protective only while energetic and quality-control capacity meet demand.

Quality control contributes to capacity. Impaired FUNDC1-mediated mitophagy aggravates mitochondrial dysfunction, ROS accumulation and degeneration, while restoring the PFKP–FUNDC1 axis improves mitophagic clearance (Fang et al., 2023). TBK1-dependent DRP1 phosphorylation suppresses excessive fission and protects mitochondrial function (Hu et al., 2023). Quality control thus determines whether chondrocytes sustain metabolic stress without accumulating dysfunctional mitochondria.

The outcome of mitophagy cannot be inferred from static markers: α-ketoglutarate enhances mitophagy, reduces ROS and preserves cartilage, and its protection is abolished by mitophagy inhibition (Liu et al., 2023), whereas Astragalus polysaccharides improved OA while suppressing NOTCH3–PINK1/Parkin–LC3-II-associated mitophagy (Xu H. et al., 2026). Although the latter is pleiotropic, together these data argue for flux-based assessment.

Reliable assessment of mitophagy requires dynamic flux measurement. Validated reporters such as mt-Keima and mito-QC visualize delivery of mitochondria to acidic lysosomal compartments, and time courses with lysosomal inhibition (bafilomycin A1, chloroquine) distinguish increased initiation from impaired degradation. Mitophagic flux - mitochondrial recognition, autophagosomal sequestration, lysosomal delivery and degradation - should therefore be quantified rather than inferred from PINK1, Parkin or LC3-II abundance.

Collectively, the consequence of FAO in OA depends on whether flux is accommodated by oxidative and quality-control capacity. Maintenance of respiratory-chain function through the SIRT3–COX4I2 axis improves bioenergetics and attenuates degeneration (Zhang et al., 2023), whereas Txnip-driven reprogramming accompanies mitochondrial dysfunction and matrix degradation (Cao X. et al., 2025). The critical variable is the dynamic balance among lipid supply, mitochondrial oxidative competence and quality control (Figure 3).

**FIGURE 3:**
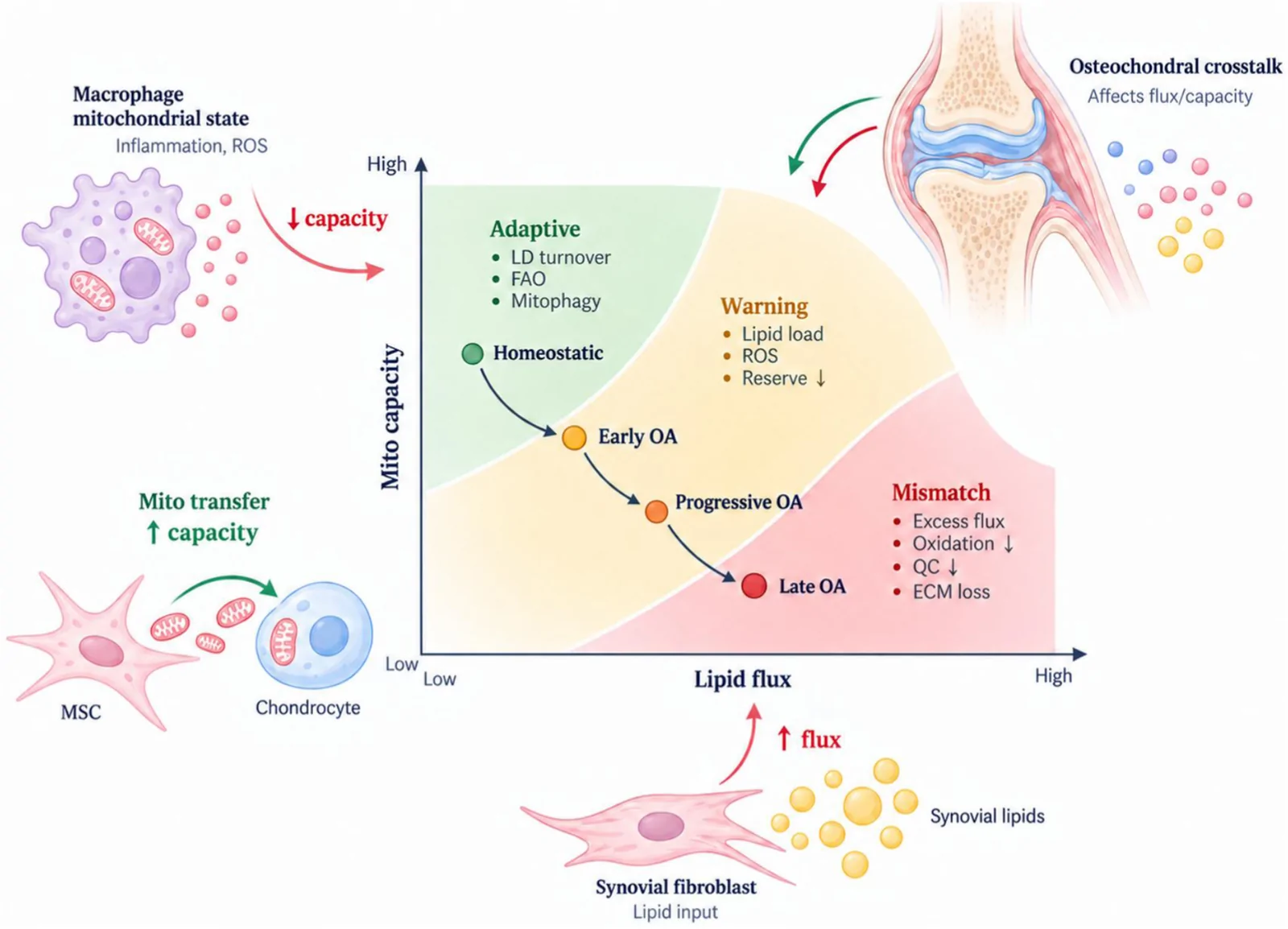
Lipid flux–capacity matching determines metabolic outcome.

## 6 Metabolic mismatch drives chondrocyte fate remodeling and whole-joint communication

Prolonged mismatch may reshape chondrocyte fate through convergent outcomes. We consider ferroptosis first, because it is the outcome most directly coupled to lipid routing and peroxidation, then senescence and matrix destabilization. LD accumulation *per se* is not pro-ferroptotic: in human cancer cell lines, DGAT-dependent PUFA sequestration into triacylglycerols suppresses ferroptosis (Lee et al., 2024), but this protection is conditional, because LD-localized FSP1 maintains neutral-lipid integrity through CoQ10 until loss of FSP1 permits oxidation of PUFA-rich triacylglycerols and LD-initiated ferroptosis (Lange et al., 2025). The ferroptotic consequence of LDs depends on PUFA partitioning and the antioxidant competence of the storage compartment, not on abundance.

In OA, ACSL4-centred remodelling links PUFA metabolism to ferroptotic injury. ACSL4 and 4-HNE increase in human and experimental OA cartilage, and ferrostatin-1 attenuates IL-1β-induced lipid peroxidation (Zou et al., 2025). Sp1-driven ACSL4 in IL-1β-treated human chondrocytes increases ROS, MDA and Fe^2+^, suppresses COL2A1/ACAN and induces MMP13, with reversal by ACSL4 silencing or ferrostatin-1 (He et al., 2023). No study has yet traced a continuous LD–PUFA–ACSL4/LPCAT3–ferroptosis axis, so susceptibility should be interpreted through lipid-pool partitioning and antioxidant capacity.

Senescence follows when quality control and redox buffering become insufficient. SIRT4 loss suppresses PINK1-mediated mitophagy, causing dysfunctional-mitochondria accumulation and senescence, reversible by SIRT4 or PINK1 restoration (Lin et al., 2024); PINK1/Parkin enhancement similarly restores membrane potential and reduces senescence and degeneration (Jie et al., 2024). Antioxidant capacity adds control: SIRT3/SOD2 activation suppresses senescence and delays OA (Zhang et al., 2024), and SIRT3/SOD2-dependent redox restoration reduces mtROS and the senescence-associated secretory phenotype (SASP)(Jiang et al., 2025). Defective mitochondrial clearance and mtROS accumulation thus reinforce senescence, potentially intersecting with lipid peroxidation, although a continuous causal circuit in OA remains unestablished.

These disturbances converge on loss of chondrocyte phenotype and matrix homeostasis. Oleic-acid accumulation in obesity-associated OA promotes CaMK2-dependent SOX9 degradation in murine chondrocytes and human cartilage explants (Li H. et al., 2026). CILP–Keap1–Nrf2 restoration increases type II/I collagen and SOX9 while suppressing lipid peroxidation and ferroptotic injury in early OA (Jia et al., 2026), and P21-dependent regulation reduces oxidative stress, promotes anabolic markers and suppresses MMP13 and ADAMTS4 (Fang et al., 2026). ECM failure is better viewed as a convergent outcome of lipid routing, mitochondrial dysfunction and redox imbalance than as a direct effect of LD abundance.

Metabolic imbalance propagates across the joint. RCAN1^+^IL-1α^+^ senescent synovial fibroblasts stabilize ATF4 and secrete saturated fatty acids that induce chondrocyte senescence, and synovium-targeted RCAN1 silencing attenuates this crosstalk (Hu et al., 2025). In synovial macrophages, pathological mechanical stress suppresses YAP–TEAD1-dependent MFN1, disrupting fusion, membrane potential and redox homeostasis, whereas YAP–MFN1 restoration reduces mtROS and catabolic signalling (Cheng et al., 2026). Macrophage-specific PGAM5 targeting likewise alters inflammatory output and protects cartilage (Liu et al., 2024). Macrophage function is thus defined by mitochondrial dynamics and redox integration rather than an M1/M2 binary.

Mitochondrial transfer extends this crosstalk. Human umbilical-cord mesenchymal stromal cells transfer mitochondria to OA chondrocytes, raising ATP production, oxygen consumption and redox resistance (Court et al., 2024), with connexin-43 (GJA1-20k) promoting contact-dependent donation (Irwin et al., 2024), and intra-articular mitochondrial transplantation attenuating degeneration (Vega-Letter et al., 2025). Conversely, mitochondrial components can also propagate pathological signals between joint compartments. Recent evidence demonstrated that infrapatellar fat pad mesenchymal stem cell-derived extracellular vesicles containing mitochondrial DNA (mtDNA) are increased in OA and can be internalized by chondrocytes, activating cGAS-STING-dependent inflammatory signaling and promoting mitochondrial stress and cartilage degeneration (Li S. et al., 2026). Thus, intercellular mitochondrial communication may exert either protective or pathogenic effects depending on the transferred cargo and cellular context. Chondrocyte–endothelial–osteoblast communication is extensive, but bone-derived lipid signals or LD–mitochondrial mechanisms remain undefined (Liu Y. et al., 2025).

Chondrocyte fate emerges from the imbalance among lipid storage and mobilization, membrane phospholipid remodelling, mitochondrial oxidation and quality control, and across the joint these processes are modulated by synovial fibroblast-derived lipids, macrophage mitochondrial state and intercellular mitochondrial transfer. Mapped onto the framework (Figure 3), synovial fibroblast-derived saturated fatty acids primarily raise the lipid-flux axis (extracellular burden and availability), whereas macrophage mitochondrial remodelling and intercellular transfer act on the mitochondrial-capacity axis (function, redox balance, resilience); osteochondral signalling may influence both through nutrient exchange and inflammation. Whether these extra-chondrocytic inputs push chondrocytes toward mismatch therefore depends on their relative magnitude, a prediction testable by manipulating each axis independently. These metabolic disturbances and their implications for chondrocyte fate remodeling and whole-joint communication in OA are summarized in Table 3.

**TABLE 3:** Lipid–mitochondrial metabolic mismatch and chondrocyte fate remodeling in osteoarthritis.

Metabolic disturbance	Major consequence	Experimental context	Evidence status	OA implication	Representative references
PUFA partitioning between TAGs and membrane phospholipids	Lipid peroxidation and ferroptotic susceptibility	Non-chondrocyte systems (human cancer cell lines)	Indirect mechanistic evidence	LD-mediated PUFA sequestration may influence ferroptosis susceptibility; however, OA-specific lipid-pool redistribution remains unresolved	(Lee et al., 2024; Lange et al., 2025)
ACSL4-dependent phospholipid remodeling	Ferroptotic injury and extracellular matrix catabolism	Human OA cartilage; IL-1β-treated human primary chondrocytes	Direct OA evidence	ACSL4 links PUFA remodeling with ferroptotic responses in OA, whereas a continuous LD–PUFA–ACSL4/LPCAT3 pathway requires further validation	(Zou et al., 2025; He et al., 2023)
Impaired mitochondrial quality control	mtROS accumulation, cellular senescence and SASP activation	Human OA chondrocytes; experimental OA models	Direct OA evidence	Defective mitophagy and impaired antioxidant systems reduce chondrocyte tolerance to metabolic stress	(Zhuang et al., 2024; Fang et al., 2023; Hu et al., 2023)
Metabolic convergence on chondrocyte phenotype	Loss of SOX9/COL2A1/ACAN expression and extracellular matrix degradation	Human cartilage explants; primary chondrocytes; experimental OA models	Direct OA evidence	Lipid remodeling, mitochondrial dysfunction and redox imbalance converge on anabolic–catabolic phenotype switching rather than LD accumulation alone	(Li H. et al., 2026; Jia et al., 2026; Fang et al., 2026)
Synovial fibroblast-derived lipid signaling	Chondrocyte senescence and catabolic remodeling	Human OA synovium; experimental OA models	Direct OA evidence	Extra-cartilaginous lipid signals increase metabolic burden on chondrocytes and primarily influence the lipid-flux axis	(Hu et al., 2025)
Macrophage mitochondrial dysfunction	Altered inflammatory output and paracrine signaling	Synovial macrophage OA models	Direct OA evidence	Macrophage mitochondrial dynamics and redox regulation influence inflammatory signaling and joint homeostasis	(Cheng et al., 2026; Liu et al., 2024)
Protective mitochondrial transfer	Increased ATP production, oxidative phosphorylation and redox resistance	MSCs; OA chondrocytes; experimental OA models	Emerging OA evidence	Intercellular mitochondrial donation may enhance recipient-cell metabolic capacity under stress conditions	(Court et al., 2024; Irwin et al., 2024; Vega-Letter et al., 2025)
Pathogenic extracellular vesicle mitochondrial signaling	mtDNA transfer, cGAS–STING activation and cartilage degeneration	IPFP-MSC-derived extracellular vesicles; OA chondrocytes	Emerging OA evidence	Mitochondrial cargo transfer may propagate inflammatory signals depending on cargo composition and recipient-cell response	(Li S. et al., 2026)
Bone–cartilage metabolic communication	Whole-joint metabolic remodeling	Human single-cell studies; OA models	Established communication; lipid mechanisms unresolved	Communication between cartilage, synovium and subchondral bone may influence both lipid flux and mitochondrial capacity, but LD–mitochondria mechanisms remain undefined	(Wang et al., 2022; Raut et al., 2025; Schurman et al., 2026)
Lipid flux–mitochondrial capacity mismatch	Ferroptosis, senescence and extracellular matrix failure	Human OA chondrocytes; experimental OA models	Integrative framework supported by multiple evidence levels	Disease progression depends on whether mitochondrial oxidative capacity and quality-control systems can accommodate altered lipid flux	(Mei et al., 2025; Cao S. et al., 2025; Liu et al., 2026; Zou et al., 2025)

## 7 Therapeutic strategies and research priorities

Therapy should restore coordination rather than directionally manipulate a single pathway. SESN2 restoration suppresses SREBP1-dependent lipogenesis (Liu Z. et al., 2025), SIRT5-dependent protein lysine malonylation controls chondrocyte metabolism under ageing and obesity (Liu H. et al., 2025), and the mitochondria-targeted antioxidant XJB-5-131 suppresses lipid peroxidation and ferroptosis (Sun et al., 2024). These are proofs of metabolic-node tractability, not of sufficiency. Beyond direct metabolic modulation, extracellular vesicle-based delivery systems have emerged as a strategy to coordinate multiple pathological processes within the OA microenvironment. A logic-based spatiotemporal dual extracellular vesicle platform has been developed to achieve sequential regulation of inflammatory responses and tissue repair, highlighting the potential of engineered vesicles to overcome the limitations of conventional single-target interventions (Li S. et al., 2024). Such approaches may provide a framework for delivering metabolic regulators, anti-inflammatory signals or organelle-modulating cargos in a temporally controlled manner.

LDs should not be depleted indiscriminately. Accumulation may reflect increased synthesis or defective turnover, as TGF-β2–TβRI–p-Smad3–FABP5 promotes neutral-lipid accumulation in chondrocytes (Wei et al., 2026), whereas APOE loss impairs lipid handling and pharmacological LXR agonism (RGX-104) partially restores homeostasis (Zhang Y. et al., 2025). For PUFAs, AMPK-driven LD dynamics redistribute LD-stored PUFAs toward mitochondria in melanoma cell lines, increasing peroxidation susceptibility (Motamedi et al., 2025), and linoleate accumulation promotes ferroptotic injury in male rats (Deng et al., 2026). The therapeutic aim is context-dependent restoration of storage, mobilization and membrane partitioning while preserving buffering.

Mitochondrial fatty-acid handling should be neither uniformly activated nor inhibited. Restricting CPT1-dependent entry improves chondrocyte fitness (Chen et al., 2024), yet sustained FAO drives acetyl-CoA accumulation, HADHA hyperacetylation and SOX9 destabilization (Mei et al., 2025), and resistin-driven CAP1–PKA–CREB signalling increases CPT1A and FAO in metabolic-syndrome synoviocytes, promoting catabolism (Ding et al., 2025). Restoring competence is preferable: cartilage-targeted SIRT3 activation enhances energy production and repair (Lu et al., 2025), fasting-induced OPTN-mediated mitophagy reduces dysfunctional mitochondria (Zhang M.-N. et al., 2025), and JP4-039 enhances PINK1/Parkin mitophagy while suppressing lipid peroxidation (Xie et al., 2025).

Static readouts cannot resolve lipid origin and fate. Time-resolved 13C pulse–chase tracing should quantify incorporation from LD-labelled triacylglycerols into acylcarnitines, acetyl-CoA and TCA-cycle intermediates (Chen S. et al., 2025), supported by LC–MS acyl-CoA profiling (Liu R. et al., 2025), pulse–chase imaging of LD-to-mitochondria trafficking (Miner and Cohen, 2024) and temporally controlled perturbation of ATGL, lipophagy and CPT1A, since LD-to-mitochondria transfer and lipophagy are differentially regulated (Xue et al., 2026).

Contact sites should be converted from descriptors into manipulable variables. Reversible split-fluorophore systems (FABCON) resolve dynamic mitochondria–LD contacts (Li X. et al., 2024), LD-membrane probes with structured illumination resolve contact remodelling and fatty-acid redistribution (Kong et al., 2024), and proximity proteomics (LipoID; PLIN3–VDAC3) links contact regulators to membrane potential, ATP, FAO and lipid profiles (Guo et al., 2026). Increased contact abundance must not be equated with increased transfer, causal tests require perturbation paired with direct lipid-transfer and flux measurements.

Lipophagy and mitophagy should be tracked together in matched systems. TfLiveDrop reports lipophagic flux *in vivo* (Gong et al., 2026), fluorescence approaches resolve sequential mitophagic intermediates (Wang Y. et al., 2025), and Rab GTPases act as selective-autophagy cues shared by lipid droplets and damaged mitochondria (Zhao et al., 2025). Whether these fluxes remain matched or dissociate across OA stages is unknown.

Whole-joint reconstruction should integrate single-cell and spatial transcriptomics with spatial lipidomics, metabolomics and isotope-resolved flux analysis across synovium, cartilage and subchondral bone. Spatial immune dynamics in synovium (Wang et al., 2025b), proteomic–metabolomic synovitis endotypes (Zhu et al., 2025), molecular gradients across the cartilage–subchondral bone interface (Schurman et al., 2026), spatial isotope deep tracing (Li et al., 2025) and lipid metabolic flux analysis (Wessendorf-Rodriguez et al., 2026) are now feasible. The goal is to locate lipid origin, recipient cells and processing pathways, which abundance maps cannot resolve, and to distinguish synthesis from uptake or inter-tissue transport.

Human validation requires multidimensional stratification: age, sex, obesity, insulin resistance and imaging or histopathological grade, supported by multimodal risk modelling (Nielsen et al., 2024) and evidence that OA is a molecular continuum with phenotype-enriched pathways (Perry et al., 2026). Mechanistic findings should then be tested in human explants and organoid models (Dönges et al., 2024) before animal and independent human validation.

## 8 Conclusion

LD–mitochondrial crosstalk in OA has no fixed direction. Its consequences depend on the coordination of lipid input, LD buffering and turnover, intracellular routing and mitochondrial oxidative and quality-control capacity. When matched, storage, mobilization and oxidation support adaptation; when flux exceeds capacity, redox imbalance, lipid peroxidation, ferroptotic susceptibility, senescence and matrix disruption follow. Therapy should restore this matching rather than target a single organelle or pathway, and research should trace lipid fate across space and time to test it.

## Glossary

4-HNE: 4-hydroxynonenal
ACC: acetyl-CoA carboxylase
ACOT12: acyl-CoA thioesterase 12
ACSL4: acyl-CoA synthetase long-chain family member 4
AMPK: AMP-activated protein kinase
APOE: apolipoprotein E
ARF1: ADP-ribosylation factor 1
ATF4: activating transcription factor 4
ATP: adenosine triphosphate
BNIP3: BCL2 interacting protein 3
CAP1: adenylyl cyclase-associated protein 1
CILP: cartilage intermediate layer protein
CMA: chaperone-mediated autophagy
CoQ10: coenzyme Q10
COX4I2: cytochrome c oxidase subunit 4 isoform 2
CPT1: carnitine palmitoyltransferase 1
CPT1A: carnitine palmitoyltransferase 1A
CREB: cAMP response element-binding protein
DGAT1: diacylglycerol O-acyltransferase 1
DNL: *de novo* lipogenesis
DRP1: dynamin-related protein 1
ECM: extracellular matrix
ESCRT: endosomal sorting complexes required for transport
ESYT1/2: extended synaptotagmin 1/2
FABCON: fluorogen-activated BiFC at contact sites
FABP5: fatty acid-binding protein 5
FAO: fatty acid oxidation
FATP4: fatty acid transport protein 4
FGF8: fibroblast growth factor 8
FGFR1: fibroblast growth factor receptor 1
FSP1: ferroptosis suppressor protein 1
FSP27: fat-specific protein 27
FUNDC1: FUN14 domain-containing protein 1
GJA1: gap junction protein alpha 1
HADHA: hydroxyacyl-CoA dehydrogenase trifunctional multienzyme complex subunit alpha
HCAR2: hydroxycarboxylic acid receptor 2
Hsc70: heat shock cognate protein 70
IL-1β: interleukin-1β
ISCU: iron-sulfur cluster assembly enzyme
LAMP2A: lysosome-associated membrane protein 2A
LD: lipid droplet
LPCAT3: lysophosphatidylcholine acyltransferase 3
LXR: liver X receptor
MDA: malondialdehyde
MFN1/2: mitofusin 1/2
MMP13: matrix metalloproteinase 13
MSC: mesenchymal stromal cell
mtROS: mitochondrial reactive oxygen species
Nrf2: nuclear factor erythroid 2-related factor 2
OA: osteoarthritis
OXPHOS: oxidative phosphorylation
PDK2: pyruvate dehydrogenase kinase 2
PDZK1: PDZ domain-containing protein 1
PFKP: phosphofructokinase platelet type
PGAM5: phosphoglycerate mutase family member 5
PINK1: PTEN-induced kinase 1
PKA: protein kinase A
PKCK2: protein kinase casein kinase 2
PKM2: pyruvate kinase M2
PLIN1/2/3/5: perilipin 1/2/3/5
PPARα: peroxisome proliferator-activated receptor α
PUFA: polyunsaturated fatty acid
RCAN1: regulator of calcineurin 1
ROS: reactive oxygen species
SASP: senescence-associated secretory phenotype
SESN2: sestrin 2
SIRT3/4/5: sirtuin 3/4/5
SOD2/3: superoxide dismutase 2/3
SOX9: SRY-box transcription factor 9
SREBP1: sterol regulatory element-binding protein 1
STAMP2: six-transmembrane protein of prostate 2
TAG: triacylglycerol
TBK1: TANK-binding kinase 1
TCA: tricarboxylic acid cycle
TEAD1: TEA domain transcription factor 1
TGF-β2: transforming growth factor-β2
TSG101: tumor susceptibility gene 101
TβRI: transforming growth factor-β receptor I
VAPB: VAMP-associated protein B
VDAC3: voltage-dependent anion channel 3
VPS13D: vacuolar protein sorting 13 homolog D
YAP: Yes-associated protein.
